# Relationship between ecological condition and ecosystem services in European rivers, lakes and coastal waters

**DOI:** 10.1016/j.scitotenv.2019.03.155

**Published:** 2019-06-25

**Authors:** B. Grizzetti, C. Liquete, A. Pistocchi, O. Vigiak, G. Zulian, F. Bouraoui, A. De Roo, A.C. Cardoso

**Affiliations:** a European Commission Joint Research Centre (JRC), Italy; b European Commission DG Environment, Brussels, Belgium; c Ludwig-Maximilians-Universitaet Muenchen, Department of Geography, Munich, Germany

**Keywords:** Ecosystem services, Ecological status, Ecosystem condition, Water Framework Directive, Sustainable Development Goals, Biodiversity, Europe

## Abstract

We quantify main ecosystem services (i.e. the contribution of ecosystems to human well-being) provided by rivers, lakes, coastal waters and connected ecosystems (riparian areas and floodplains) in Europe, including water provisioning, water purification, erosion prevention, flood protection, coastal protection, and recreation. We show European maps of ecosystem service capacity, flow (actual use), sustainability and efficiency. Then we explore the relationship between the services and the ecosystem condition at the European scale, considering the ecological status of aquatic ecosystems, reported under the EU Water Framework Directive, as a measure of the ecosystem integrity and biodiversity.

Our results indicate that a higher delivery of the regulating and cultural ecosystem services analysed is mostly correlated with better conditions of aquatic ecosystems. Conversely, the use of provisioning services can result in pressures on the ecosystem. This suggests the importance of maintaining good ecological condition of aquatic ecosystems to ensure the delivery of ecosystem services in the future. These results at the continental scale, although limited to the ecosystem services under analysis, might be relevant to consider when investing in the protection and restoration of aquatic ecosystems called for by the current EU water policy and Biodiversity Strategy and by the United Nations Sustainable Development Goals.

## Highlights

• We quantify main ecosystem services of rivers, lakes, coastal waters in Europe.
• We show European maps of water ecosystem service capacity, flow and sustainability.
• We explore the link between ecosystem services and conditions (ecological status).
• Higher ecosystem service delivery is mostly correlated to better ecological status.
• The results show the relevance of protecting and restoring aquatic ecosystems.

## Graphical abstract

**Figure:**
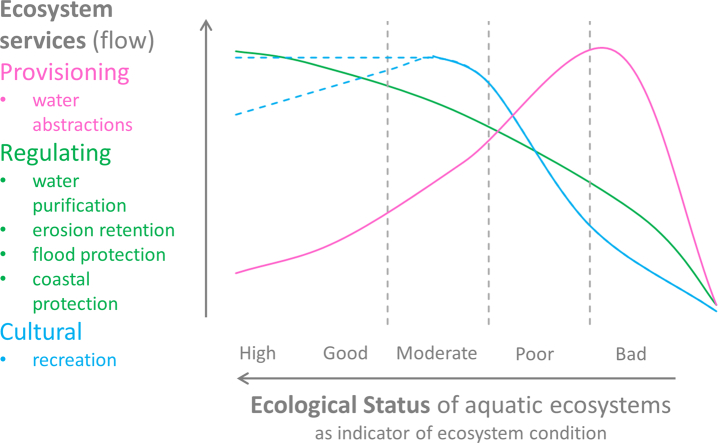

## 1 Introduction

In 2010 under the international Convention on Biological Diversity (CBD, 1992) the parties adopted a Strategic Plan including 20 targets (the Aichi Biodiversity Targets) to “take effective and urgent action to halt the loss of biodiversity in order to ensure that by 2020 ecosystems are resilient and continue to provide essential services, […] securing the planet's variety of life, and contributing to human well-being, and poverty eradication”.

The principles of the CBD are also embedded in the United Nations Sustainable Development Goals (SDG) to 2030 (United Nations, 2016). In particular, for water ecosystems, SDG6 supports the protection and restoration of water-related ecosystems, SDG15 calls for the conservation, restoration and sustainable use of terrestrial and inland freshwater ecosystems and their services, promoting the integration of ecosystem and biodiversity values into national and local planning, and SDG14 aims at the conservation and sustainable use of coastal and marine resources.

In 2011, to put into effect the commitments taken under the CBD, the European Union adopted the EU Biodiversity Strategy to 2020 (European Commission, 2011), setting out 6 targets to halt the loss of biodiversity and ecosystem services in the EU by 2020. The targets aim to conserve and restore nature, enhance ecosystems and their services by establishing green infrastructure and restoring degraded ecosystems, integrate biodiversity into the development of agriculture, forest and fisheries policies, combat invasive alien species, and avert global biodiversity loss.

The objectives of the Biodiversity Strategy are equally relevant to the EU water policy, whose purpose is to protect and enhance EU water resources and aquatic ecosystems. The Water Framework Directive (WFD, Directive 2000/60/EC) aims to achieve and maintain a good ecological status for all EU rivers, lakes, groundwater, coastal and transitional waters, and to manage water resources in a sustainable way through the implementation of river basin management plans (RBMP). Similarly, the goal of the Marine Strategy Framework Directive (MSFD, Directive 2008/56/EC) is to protect marine waters and to reach a good environmental status for the entire EU marine environment. The EU water policies recognize the role of water resources to sustain economic activities and human well-being as well as the impact of pressures that socio-economic drivers put on aquatic ecosystems, and call for the protection of aquatic ecosystems and the sustainable management of water resources.

The focus on ecological status introduced by the WFD in 2000 was pioneering (Carvalho et al., 2019); it established the protection and restoration of aquatic ecosystems condition. The notion of ecosystem condition adopted in policies setting conservation and sustainability targets for ecosystems, like in the case of the WFD, is multifaceted, including aspects of integrity, health and functioning, stability and resilience, capacity to maintain ecological functions and delivery ecosystem services (Roche and Campagne, 2017). Ecosystem condition (i.e. “the physical, chemical and biological condition or quality of an ecosystem at a particular point in time”) can be linked to well-being through ecosystem services, as the condition affects the delivery of multiple ecosystem services (Maes et al., 2018).

Quantifying and valuing ecosystem services (i.e. direct and indirect contributions of ecosystems to human well-being, TEEB, 2010) could be useful to recognize all the benefits that humans receive from nature, offering stronger arguments to protect and restore ecosystems (Guerry et al., 2015), and helping achieving sustainability goals. To this purpose the ecosystem service approach, by considering the multiple benefits that people receive from aquatic ecosystems, can highlight trade-offs and also hidden benefits that are often unaccounted for in traditional cost-benefits analysis (Brauman et al., 2007; Liquete et al., 2016a). Including all ecosystem services provided by aquatic ecosystems could also justify the cost of their protection and restoration (Pouso et al., 2018a). However, it is important to notice that the economic valuation of ecosystem services does not necessarily ensure the conservation of biodiversity, especially when human management, market incentives and distribution of benefits among stakeholders hamper synergies between biodiversity and ecosystem processes, services and goods (Adams, 2014). All these aspects are of great interest for the implementation of the water policy in the EU, where the application of RBMP involves substantial costs (Vlachopoulou et al., 2014; Grizzetti et al., 2016a; Stosch et al., 2017).

In Europe the Mapping and Assessment of Ecosystems and their Services (MAES) Working Group was established to support the implementation of the EU Biodiversity Strategy with the aim of developing common methodologies for mapping and assessing ecosystem services (Maes et al., 2016). In addition, several EU funded research projects have been working on the mapping and the operationalisation of the ecosystem services concepts for improved management of water, land and urban areas (OpenNESS, 2017; OPERA, 2017). More specifically on water ecosystems, the EU FP7 project MARS analysed the impacts of multiple stressors on the ecological status of European aquatic ecosystems (rivers, lakes, groundwater and coastal water) and on their provisioning of ecosystem services (Hering et al., 2015). These projects have produced valuable mapping of terrestrial ecosystem services (Maes et al., 2015), tools and real case ecosystem services assessments (Dunford et al., 2018). However, research gaps remain in the quantification of riverine ecosystem services (Hanna et al., 2018), and an assessment of ecosystem services provided specifically by aquatic ecosystems at the European scale has not been developed yet.

Furthermore, it remains critical to show evidence of the link between the ecosystem condition and the delivery of ecosystem services at the large scale (Ricketts et al., 2016). The relationships between biodiversity and ecosystem functions and between biodiversity and ecosystem services are complex (Duncan et al., 2015). Recent studies have focused on the connection between natural capital and ecosystem services in terrestrial ecosystems (Smith et al., 2017) and between conditions in estuaries and cultural ecosystem services (Pouso et al., 2018b). Understanding the links between pressures, ecological condition, and delivery of ecosystem services is crucial for the sustainable management of water resources and aquatic ecosystems and can shed light on the effects of future change on the provision of benefits for the society.

The objective of this study was to quantify and map the ecosystem services delivered by aquatic ecosystems (rivers, lakes, transitional and coastal waters and the connected ecosystems riparian areas and floodplains) at the European scale, and analyse their relationship with the ecosystem condition. The assessment was based mainly on a biophysical valuation of the ecosystem services (Pascual et al., 2017). The paper is organised as follows. After describing the methodological approach, we present the assessment of ecosystem services related to aquatic ecosystems at the European scale, showing European maps of the services. Then we analyse the relationships between the ecosystem condition and the delivery of services, discussing the links between pressures, ecological status and ecosystem services, and the implications for policy implementation.

## 2 Material and methods

### 2.1 Assessment of European water ecosystem services

We quantified major provisioning, regulating and cultural ecosystem services related to aquatic ecosystems at the European scale, including water provisioning, water purification, erosion prevention, flood protection, coastal protection, and recreation (Table 1, for the service erosion prevention the assessment covered the Danube River Basin, not the whole Europe). These services are provided by aquatic ecosystems, such as lakes, rivers, groundwater, transitional and coastal waters, and by ecosystems at the land and water interface, such as riparian areas, floodplains and wetlands (Table 1). For the classification of the services we followed the CICES classification v4.3 (https://cices.eu). Our analysis is not exhaustive of all ecosystem services provided by aquatic ecosystems. The assessment was limited by the data availability at the European scale and our possibility to compute quantitative indicators (Table 1).

**Table 1:**
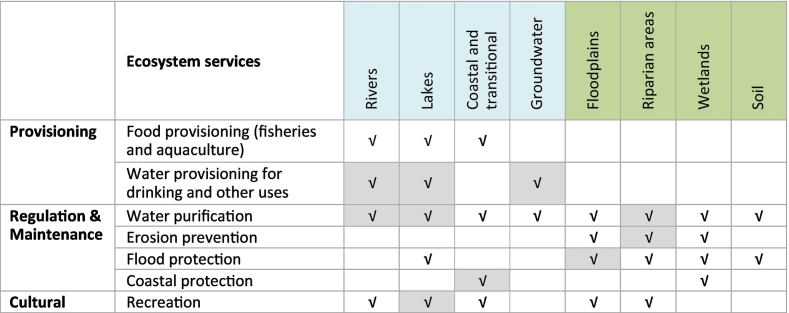
Ecosystem services considered in the study (highlighted in grey) and relevance/presence of the services per ecosystem type (√). For the service erosion prevention the assessment covered the Danube River Basin, not the whole Europe.

**Fig. 1:**
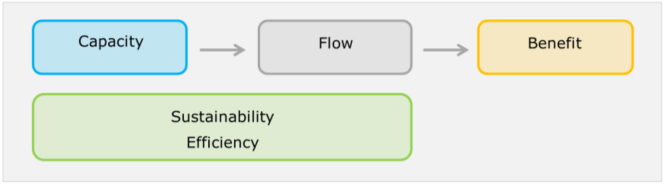
Conceptual framework to classify indicators of water ecosystem services.

For each ecosystem service we quantified proxies/indicators that are able to describe the different aspects of the service, considering indicators of the service capacity, flow, sustainability, efficiency, and when possible of the benefits, according to the conceptual framework discussed in Grizzetti et al. (2016b) (Fig. 1). The *capacity* refers to the natural potential of the ecosystem to provide the service. The *flow* is the actual use of the service. Indicators of *sustainability* of the service inform on the sustainable use of the service, considering capacity and flow together. Where the capacity for some services is unknown or unaccountable, indicators on the efficiency of the process responsible of the service can inform on the service *efficiency*. *Benefits* are associated with human well-being and value system (Fig. 1). Distinguishing between the different typologies of indicators allows to correctly identify the information provided by each indicator, supporting the analysis of the relationship between pressures, ecosystem condition and ecosystem services delivery.

(Adapted from Grizzetti et al., 2016b, Fig. 4).

The proxies/indicators to quantify water ecosystem services at the European scale were selected among those proposed in Grizzetti et al. (2016b) based on the review of international studies (Egoh et al., 2012; Layke et al., 2012; Liquete et al., 2013a; Russi et al., 2013; Maes et al., 2016) and considering the feasibility to estimate them at the European scale. The indicators were computed using European data and models, according to best available data for the period 2005–2010. The quantification of ecosystem services indicators was performed using the spatial units most relevant for the River Basin Management Plans, i.e. small catchments (average size 180 km^2^) for rivers, riparian areas and floodplains (for the number and area distribution of the catchments adopted in this study see Supplementary material S1); water body for lakes (geographical information from Ecrins, EEA, 2012b); and areas units of average coastal length of 30 km for coastal water.

In the following part we describe the ecosystem services analysed in this study. The proxies/indicators adopted are summarised in Table 2. In Supplementary material we provide a short glossary of terms (Supplementary material S2) and information on the ecosystem service fish provisioning (Supplementary material S3).

**Table 2:** Proxies/indicators to quantify ecosystem services at the European scale adopted in this study.

Ecosystem services	Natural capacity	Service flow	Sustainability or efficiency	Benefit
Water provisioning (for drinking and non-drinking)	• Total renewable water	• Water demand	• Water Exploitation Index (sustainability)	
Water purification	• Natural areas in floodplains	• Nitrogen retention	• Ratio of nitrogen retained vs. total input to water body (efficiency)	
Erosion prevention	• Density of vegetated riparian land	• Sediment retention in riparian land	• Ratio sediment retention in riparian land vs. total input to water body (efficiency)	
Flood protection	• Natural areas in floodplains	• Water volume retained for a flood with 200 years return time		
Coastal protection	• Protection capacity of natural systems	• Protection supply		• Human demand for coastal protection
Recreation and tourism	• Recreation potential	• Recreation opportunity spectrum		

#### 2.1.1 Water provisioning

Fresh water is a fundamental service that nature provides to humans. Groundwater, rivers and lakes can be sources of clean water for drinking purposes and domestic uses, and they provide water for economic activities, such as industry, energy production, irrigation and livestock. Water provisioning refers to the water that is abstracted from the water bodies, and can eventually be released back to the water system.

##### Natural capacity

The total renewable water (m^3^/y, long term average of the stream flow plus net groundwater recharge) that is naturally produced by a river basin indicates the capacity of the system to provide water. It depends on climate, geology, topography, soil and vegetation characteristics of the river basin. We used a European map of total renewable water estimated with a Budyko (1974) approach, as in Pistocchi et al. (2019). The natural capacity includes the water to be allocated for human uses and for supporting the aquatic ecosystems (i.e. environmental flow requirements), as in Karabulut et al. (2016).

##### Service flow

The actual use of freshwater by humans is quantified by the annual water abstractions for different uses (m^3^/y), which include: drinking purposes, domestic use, industry, energy production, irrigation and livestock. Each use has specific requirements on the quality of water and the temporal availability. We assumed that water demands are a good proxy for water abstractions. In Europe, the quantification and mapping of water demand is based on statistics reported by countries to EUROSTAT and FAO (Vandecasteele et al., 2014; Mubareka et al., 2013; De Roo et al., 2012). We computed a European map of total water demand.

##### Sustainability

The sustainability of water provisioning can be assessed by indicators of water scarcity that combine the natural water availability with the amount of human abstractions in a river basin. The Water Exploitation Index (WEI) has been applied in studies on water scarcity at the European scale (De Roo et al., 2012). It is computed as the ratio between total water abstractions (m^3^, considering all uses), and the total available water (m^3^). WEI is expressed as a fraction. We used a European map of WEI computed considering the total available water estimated at the catchment level by the Budyko approach.

#### 2.1.2 Water purification

Water purification indicates the removal of pollutants from water that is mediated by microorganisms and other ecosystem processes such as filtration, sedimentation and chemical processes. In large scale assessments the nitrogen retention has been used as proxy for water purification service (Liquete et al., 2015; Sharp et al., 2015; La Notte et al., 2015). Nitrogen retention is a proxy of particular relevance considering the level that nitrogen pollution has assumed at the global scale (Rockström et al., 2009).

##### Natural capacity

In aquatic ecosystems nitrogen retention can be temporal, related mainly to algae and plant uptake, or permanent when nitrogen is lost to the atmosphere by the process of denitrification operated by bacteria (Saunders and Kalff, 2001). Denitrification takes place in anoxic conditions where nitrate and electron donors are simultaneously available. Besides in soils and wetlands, these conditions occur in groundwater, hyporheic zones, riparian sediments, bottom waters and sediments of lakes and estuaries, and in the water column of suboxic river reaches (Seitzinger et al., 2006). Overall, the interfaces between land and water are very actives zones for biogeochemical processes and denitrification. The actual capacity of the ecosystem to remove nitrogen, or pollutants, cannot be measured. For large scale assessments, we consider that spatial data on area occupied by wetlands, riparian vegetation, rivers and lakes can be used as proxies to map the presence of the service. We used the fraction of land occupied by natural areas in floodplains by Pistocchi et al. (2015), based on floodplains as delineated by Weissteiner et al. (2016) combined with the land cover data of the Corine Land Cover map of 2012 (CLC, 2012) (for a discussion of the role of natural floodplains in water self-purification and flood protection see Kiedrzyńska et al., 2015).

##### Service flow

Generally the amount of nitrogen removed by water ecosystems is estimated as the difference between input and measured output, or by means of biogeochemical models that consider the water cycle and nutrient processes. We used the nitrogen retention in surface waters estimated by the GREEN model (Grizzetti et al., 2012) at the European scale.

##### Efficiency

We calculated the nitrogen retention efficiency as the quantity of nitrogen removed by the water system divided by the total amount of nitrogen that enters the water system, both estimated by the model GREEN at the spatial units of catchments (Grizzetti et al., 2012).

#### 2.1.3 Erosion prevention

Sediment retention is the service provided by vegetation mitigating the adverse impact of incoming sediments on the freshwater body. Riparian areas are active zones for biogeochemical processes and hydrological connectivity. They are crucial to many ecosystem services. Besides nursery habitat and pollution retention, riparian areas reduce sediment fluxes in the freshwater systems, by trapping sediments generated in the basin and stabilizing the stream banks (Stutter et al., 2012; Dosskey et al., 2012). Information on erosion prevention was available only for the Danube river basin, which represents 21% of the area covered by the present European study (Supplementary material S1).

##### Natural capacity

Natural capacity for erosion prevention can be expressed in terms of riparian land area per unit of stream length (km^2^/km). For this study we used the riparian land density estimated for the Danube river basin by Vigiak et al. (2016), based on the riparian land mapped by Clerici et al. (2013).

##### Service flow

The service flow of sediment retention can be expressed as sediment load removal afforded by riparian land, namely as the difference of mean annual sediment yields (t/km^2^/y) that cross any given reach in the absence and in the presence of riparian land. The service flow should consider both the process of trapping of sediments generated by hillslope erosion and the prevention of streambank erosion. Sediment yields can be estimated by process-based models that simulate sediment fluxes in the landscape, e.g. hillslope erosion, the sediment trapping in the riparian areas before reaching the river network, sediment transport in rivers, and streambank erosion in reaches. The hydrological model SWAT (Arnold et al., 2012) has been used in spatial analysis of sediment transport in river basins (Gassman et al., 2014). For this study we used the assessment of sediments removal by riparian land in the Danube river basin based on the SWAT model by Vigiak et al. (2016).

##### Efficiency

Under high sediments load the capacity of riparian land to trap sediments can decline progressively (Dosskey et al., 2010). At the large scale the sustainability of the erosion prevention service cannot be assessed with field measurements. However, the efficiency of sediments removal by riparian land can be estimated by modelling outputs, as the ratio between the sediments retained by the riparian land (service flow) and the total amount of incoming sediments in the absence of riparian land. For this study we used the estimation of the sediment removal efficiency (fraction) in the riparian land of the Danube river basin, based on the SWAT model by Vigiak et al. (2016).

#### 2.1.4 Flood protection

Flood protection is the service provided by floodplains that can store and slow down the water flow during floods events.

##### Natural capacity

The capacity of the ecosystem to protect against floods is represented by the connected natural areas in floodplains, where water can expand and be stored, slowing down the high flow peaks. The European assessment of the fraction of natural areas in floodplains was taken from the study of Pistocchi et al. (2015), which used the floodplains delineated by Weissteiner et al. (2016) combined with the land cover data of the Corine Land Cover map of year 2012 (CLC, 2012).

##### Service flow

The protection against floods is represented by the reduction of flood peak discharges. The actual service flow can be described as the flood attenuation granted by floodplains in natural conditions during a flood event. This attenuation can be computed overlaying the information on land cover with the flood risk maps for given return times (see the proposed methodology in Supplementary material S4). For this study, we estimated the flood attenuation (m^3^/s) considering floods with return time of 200 years (provided by Alfieri et al., 2014), and artificial flood defence of 100% in urban areas and 50% in agricultural areas.

##### Efficiency

As an indicator of the efficiency of the ecosystem service of flood attenuation we computed the ratio between the flood attenuation and the maximum flow peak, considering floods with return time of 200 years (provided by Alfieri et al., 2014).

#### 2.1.5 Coastal protection

Coastal protection is the role that ecosystems play in reducing the impacts of coastal hazards such as inundation and erosion from waves, storms surge or sea level rise. The service includes all habitat types but excludes human-made structures.

Liquete et al., 2013b, Liquete et al., 2016b developed specific indicators for the capacity, flow and benefit of this service in Europe, and tested them for the Euro-Mediterranean zone. Here, we applied a new update of the coastal protection indicators for all EU-28. Data sources include hydrodynamic models or observations, habitat and land-use maps, and geographical and sociological characteristics. The study area is the coastal zone potentially affected by extreme hydrodynamic conditions (as defined in Liquete et al., 2013b). More information about these indicators can be found in the supplementary information of Liquete et al. (2016b).

##### Natural capacity

The indicator of coastal protection capacity (CPcap) represents the natural capacity that coastal ecosystems possess to attenuate waves and currents or to harden coasts. The methodology estimates a protection score (i.e. the level of protection provided by each natural feature) of each morpho-sedimentological feature, seabed habitat and land cover type present in the coastal zone. CPcap integrates quantitatively data about coastal geomorphology, slope, presence and distribution of both emerged and submerged habitats.

##### Service flow

The level of supply of coastal protection (CPsup) integrates CPcap with an indicator of exposure, estimating the excess of capacity over exposure. The natural exposure of a coastal zone is based on the oceanographic conditions, namely wave regime, storm surge, tide and relative sea level rise. Values close to −1 point to deficient natural capacity for the existing oceanographic conditions while values close to +1 indicate enough capacity to deal with the natural exposure.

##### Benefit

The social benefit of this service can be reflected by the estimated human demand for protection in the coastal area (CPdem). This indicator is based on the presence of residents and assets in the coastal zone, in particular census of population, artificial surface and cultural sites (the latter with slightly less importance in the final calculation).

#### 2.1.6 Recreation

Nature-based outdoor recreation concerns outdoor activities generating benefits in daily life, spanning from having a walk in the closest green urban area, to a short bike ride in a local natural park, to a day trip (<100 km travelling distance) with the sole purpose of experiencing nature. All ecosystems are considered to be potential providers of the recreation service, irrespective from their conservation status, though the range of provision changes according to ecosystem characteristics and people's preferences and behaviour. In this study we used the results provided by Zulian et al. (2013) and Paracchini et al. (2014), who developed a model to assess nature-based outdoor recreation in Europe. A recent update of such model (including improved parametrization and data sources) has been used here to extract the following indicators (Liquete et al., 2016b).

##### Natural capacity

The Recreation Potential Indicator (RPI) estimates the capacity of ecosystems and natural features to support nature-based recreation activities. RPI integrates four components: suitability of land to support recreation (land use/land cover classes scored for recreation); natural features (presence and typology of natural protected areas, presence of grassland in agricultural areas); water (distance from water bodies and coast, presence of natural riparian areas, geomorphology of coast); and presence of green urban areas.

##### Service flow

The Recreation Opportunity Spectrum (ROS) combines the RPI with a proximity map. The latter integrates distance from residential areas and distance from roads. Still, this indicator does not account for the actual visitors' flow, since that information is not available at continental scale. ROS values are divided into 9 qualitative classes which combine different levels of service provision and remoteness (from 1, i.e. low provision not accessible, to 9, i.e. high provision and easily accessible).

### 2.2 Relationship between ecosystem services and ecological condition in EU water bodies

We explored the relationship between the condition of the water ecosystem and its delivery of each ecosystem service, considering all indicators assessed in the study. We performed the analysis at the European scale using the data on ecological status reported by EU Member States under the Water Framework Directive first reporting period (2004–2009) (EEA, 2012a). The WFD establishes that EU “Member States shall protect, enhance and restore all bodies of surface water […] with the aim of achieving good surface water status” (WFD, Article 4), and lays down the criteria for evaluating the ecological status of water bodies (WFD, Annex V). The ecological status is defined in five categories: high, good, moderate, poor and bad. Quality elements for the classification of ecological status includes: 1) biological elements (composition and abundance of phytoplankton, aquatic flora, benthic invertebrate fauna, fish fauna); 2) hydromorphological elements supporting the biological elements (hydrological regime and morphological conditions); and 3) chemical and physico-chemical elements supporting the biological elements. The ecological status is established for each water body at the local scale by the regional water authorities. The different methodologies applied to establish ecological status were inter-calibrated to ensure the comparability and coherence of the assessment across the EU (Poikane et al., 2015; Birk et al., 2012). The data on ecological status collected by the EU Member States represent a consistent dataset on the ecological condition of water bodies across Europe. However, these data have some limitations, as in some regions they were based on physico-chemical conditions, or on expert judgment, or not all the biological elements were used.

Integrating information on the abundance and composition of different organisms of the food web and on the habitat and chemical conditions of the aquatic environment, the ecological status can represent an integrative measure of the condition of the water body. Indeed, its definition includes different aspects of ecosystem integrity, biodiversity, functioning and quality (see the examples of potential indicators suggested in Table 1 of Roche and Campagne, 2017).

For this study valid information (centroid and ecological status) for 79,630 water bodies across the EU was available. As a proxy indicator of the ecological condition that could be representative at the scale of the assessment, we used the most frequent class (mode) of ecological status in the spatial units of the assessment, i.e. catchments, lakes and coastal units.

For the analysis we considered the distribution of the ecosystem services indicators (Table 2) per classes of ecological status. We performed Kruskal-Wallis rank tests on the class medians to detect significant differences between the five classes and Jonckheere-Terpstra tests to identify positive or negative relationships between the delivery of ecosystem service and the ecological status.

## 3 Results

### 3.1 Mapping and assessment of European water ecosystem services

#### 3.1.1 Water provisioning

**Fig. 2:**
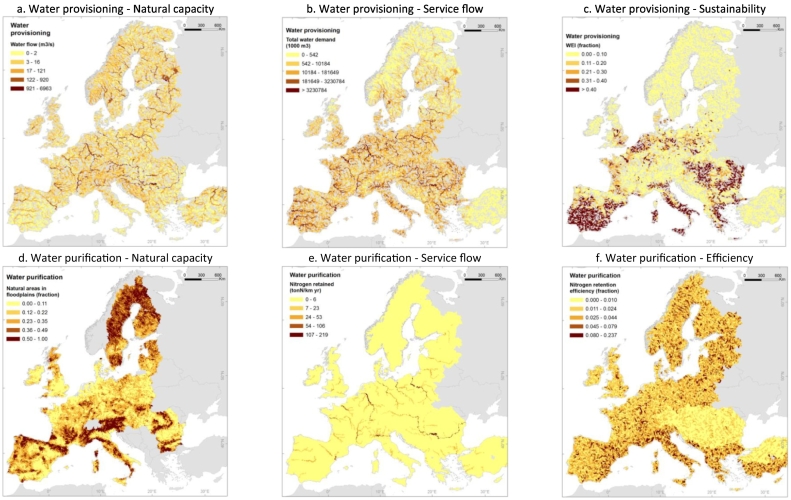

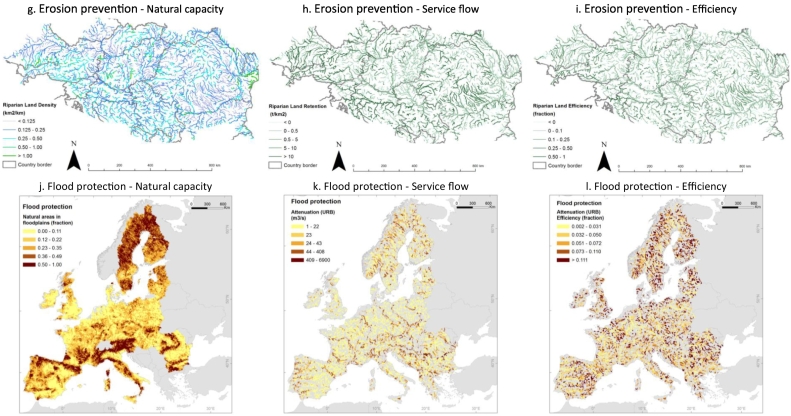

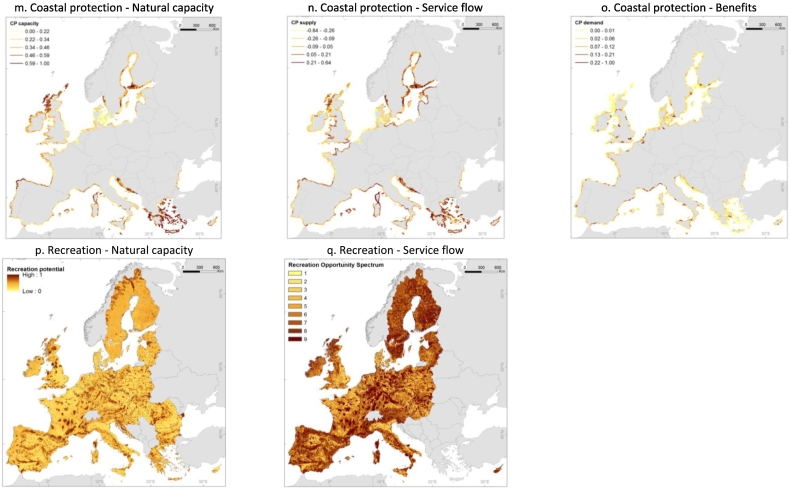
European maps of the ecosystem services. *Water provisioning* in rivers and lakes: a. Natural capacity (total renewable water); b. Flow (total water demand, De Roo et al., 2012; Vandecasteele et al., 2014; Mubareka et al., 2013); c. Sustainability (Water Exploitation Index, WEI). *Water purification* in rivers and lakes (data refer to year 2005): d. Natural capacity (areas in floodplains, Pistocchi et al., 2015); e. Nitrogen retention in surface waters (Grizzetti et al., 2012); f. Nitrogen retention efficiency (based on the results of the GREEN model (Grizzetti et al., 2012)). *Erosion prevention* (data refers to annual means for the period 1995–2009) by riparian land in the Danube river basin (800,000 km^2^): g. Natural capacity (riparian land density, Vigiak et al., 2016 based on land map of Clerici et al., 2013); h. Flow (sediment removal by riparian land, Vigiak et al., 2016); i. Efficiency (efficiency of sediments removal, Vigiak et al., 2016). *Flood protection* in floodplains: j. Natural capacity (natural areas in floodplains, Pistocchi et al., 2015, it is the same as Fig. 2d); k. Flow (flood attenuation); l. Efficiency (efficiency of flood attenuation). *Coastal protection* (CP) in coastal areas (data are based on Liquete et al., 2016b and refer to year 2010): m. Natural capacity (coastal protection capacity); n. Flow (coastal protection supply); o. Benefits (coastal protection demand). *Recreation* (data are based on Zulian et al., 2013 and Liquete et al., 2016b and refer to year 2010): p. Natural capacity (Recreation Potential indicator); q. Flow (Recreation Opportunity Spectrum indicator, ROS) ROS values: 1 = Low provision not easily accessible, 2 = Low provision accessible, 3 = Low provision easily accessible, 4 = Medium provision not easily accessible, 5 = Medium provision accessible, 6 = Medium provision easily accessible, 7 = High provision not accessible, 8 = High provision accessible, 9 = High provision easily accessible.

Europe is naturally rich in water resources, especially in its Northern, Western and Central parts, while the Mediterranean region features lesser availability due to climatic conditions. The European map of total renewable water is presented in Fig. 2a. Water demand is intense across the continent; it is related to high population density in some areas, energy production and economic activities, and irrigation of crops in water scarce regions that are also intensive agricultural areas (Fig. 2b). As a result of water abstractions, even water rich areas are under pressure (WEI values between 0.20 and 0.30), such as in Northern Europe and in the Po valley in Italy, while the worst conditions are highlighted e.g. in Southern European regions (WEI values > 0.30), see Fig. 2c.

#### 3.1.2 Water purification

It is not possible to compute the total capacity of the ecosystem to retain nitrogen but it can be assumed that it depends on the extent of the ecosystems where the retention processes can take place, such as in riparian areas in floodplains (Fig. 2d), which are active zones of nitrogen removal (Kiedrzyńska et al., 2015). Fig. 2e shows the nitrogen retention estimated in this study using the model GREEN (Grizzetti et al., 2012). Nitrogen retention is higher in large rivers, which receive more pollution, and lakes with longer residence time (Fig. 2e), but the efficiency decreases from upstream to downstream rivers (Fig. 2f). Indeed, scientific studies have shown that increasing the amount of nitrogen discharged in the water body lowers the efficiency of nitrogen removal (Mulholland et al., 2008). Increasing nitrogen load to water bodies can foster the process of eutrophication, with a consequent reduction of habitat and biodiversity, decreasing the ability of the ecosystem to uptake nitrogen (Cardinale, 2011).

#### 3.1.3 Erosion prevention

The density of riparian land per km of river estimated in the Danube river basin is shown in Fig. 2g. The analysis for the Danube river basin based on the SWAT model has estimated that on average natural riparian lands retain 0.86 ton/km^2^/y of sediments (Fig. 2h). The filtering process is more efficient in lower Strahler's order reaches (upstream catchments). The median filtering efficiency decreased from 17% in reaches of Strahler's order 1 to 5% in reaches of Strahler's order larger than 3 (middle and downstream catchments) (Fig. 2i). At the same time, streambank protection service is important in reaches of higher Strahler order and in regions of high stream power, like in the Alps (Fig. 2i).

#### 3.1.4 Flood protection

The indicators of flood protection service proposed in this study, i.e. the attenuation of flow peak by natural areas in floodplains, combines information on the floodplain capacity to store water (approximated by the natural areas in floodplains, Fig. 2j) and the volume of flood with a certain return time, taking into account the land cover change (specifically, the presence of artificial and agricultural lands). The assessment of flood protection highlights the widespread presence of this service across Europe, with higher rates in floodplains where flood risk is higher and natural vegetation prevails on urbanization (Fig. 2k). The efficiency of the service informs on the ratio between flood attenuation and maximum flow peak (Fig. 2l).

#### 3.1.5 Coastal protection

The maximum average values of the indicator for natural capacity of coastal protection are present in Malta and Greece (Fig. 2m). The service flow of coastal protection shows maximum values in Latvia, while most negative values concentrate around the North Sea, Northeast Atlantic and North and West Adriatic Sea (Fig. 2n), indicating a potential unsustainable situation between the ecosystem service capacity and the exposure. We remind that human-made protection works (e.g. hard defence structures, designed flooding areas), especially developed in the shores of the North Sea, are not reflected in this analysis. Belgium shows both the maximum exposure and the maximum values of coastal protection demand (Fig. 2o), pointing to one of the riskiest contexts. The capacity of natural habitats to reduce the impacts of coastal hazards should be analysed through time since it tends to decrease in recent decades driven by land use and shoreline changes (Liquete et al., 2016b). The possible decline of coastal protection capacity and actual supply (service flow indicator in our analysis) combined with an expected growth of demand should be of concern for coastal communities.

#### 3.1.6 Recreation

The outdoor recreation is measured here in terms of extent and quality of citizens' access to nature, considering all ecosystems as potential providers of the service but highlighting the attraction of water bodies for recreation. Mapping of the Recreation Potential illustrates that the service capacity in Europe is relatively high. According to Paracchini et al. (2014), almost half of the territory is classified in the highest classes of recreation provision, but the spatial distribution of such potential is uneven. Based on the new results presented here, all EU countries show an average Recreation Potential below 0.4, with Slovenia and Croatia getting the maximum values (Fig. 2p). The natural capacity transforms into a service flow when people can reach sites for outdoor recreation, as reflected by Recreation Opportunity Spectrum indicator. Again, the service flow in Europe is relatively high, with 33% of the territory under “High provision easily accessible” and 10% under “High provision accessible” classes (Fig. 2q). We must note that only 5% of Europe is not easily accessible in this analysis.

### 3.2 Relationship between ecosystem condition and ecosystem services

We performed a systematic analysis of the relationship between the data on the ecological status and water ecosystem services distinguishing between the capacity, flow, efficiency, sustainability and benefit of the services.

**Fig. 3:**
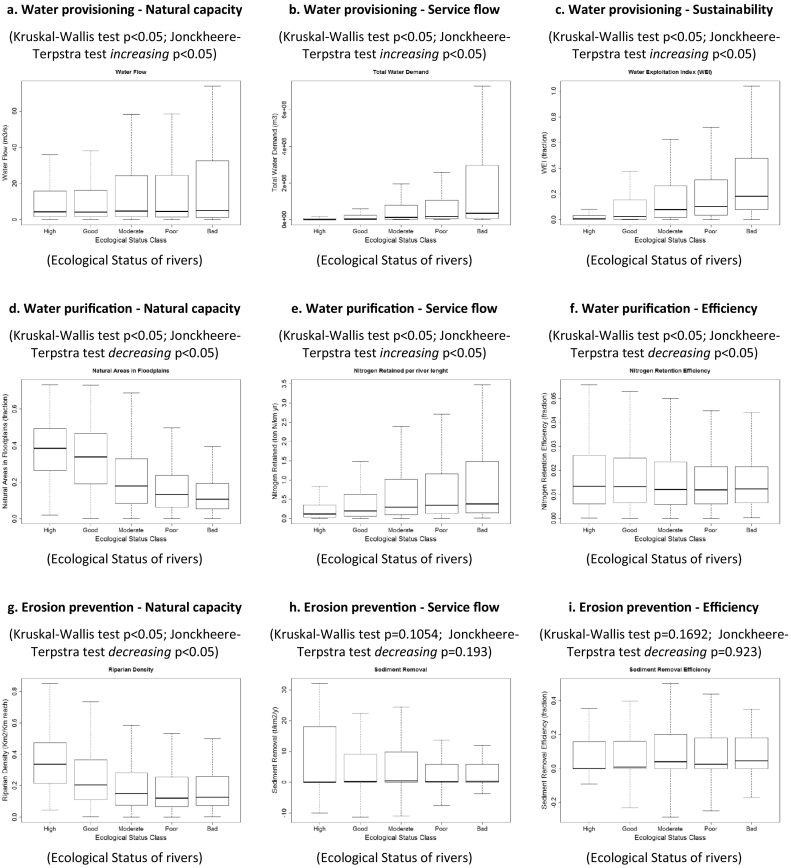

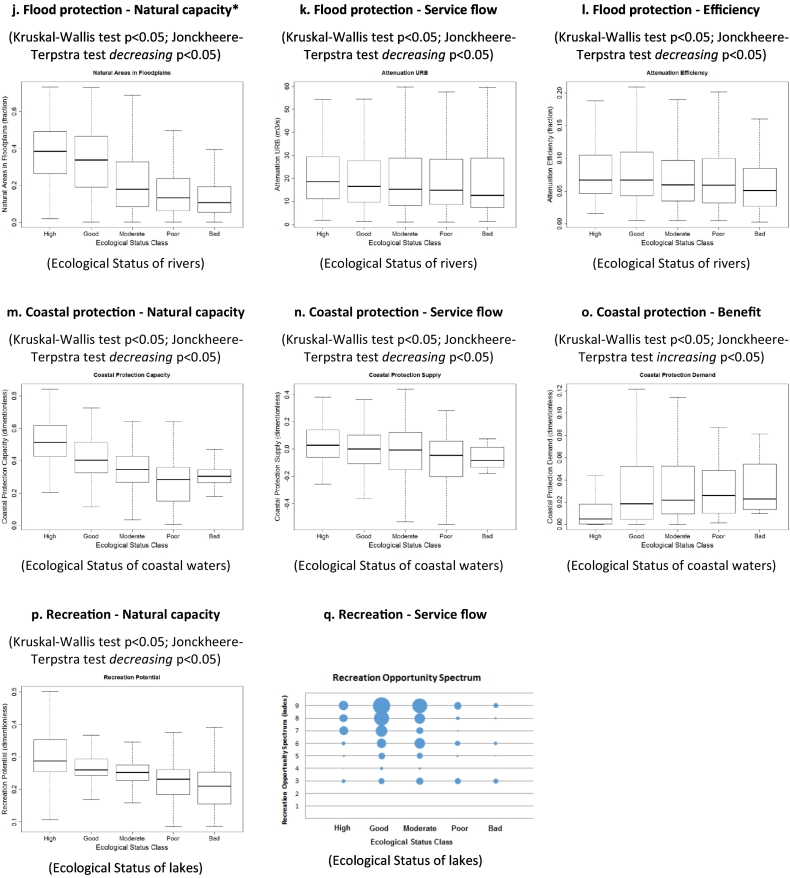
Relationship between the indicators of the ecosystem services analysed in this study and the proxy of the ecological status for European aquatic ecosystems. p indicates the significance of the Kruskal-Wallis and the Jonckheere-Terpstra statistical tests. (*Fig. 3d is the same as 3j).

Our results show that water provisioning is higher in catchments where rivers are in moderate to bad ecological status, which are also the areas where water stress is prominent (higher values of WEI) (*p* < 0.05, Fig. 3a–c). Similarly, water purification (nitrogen retention) increases in rivers in poorer conditions, but the natural capacity to retain nitrogen and the efficiency of the process show an opposite relationship, being greater in ecosystems in better ecological status (*p* < 0.05, Fig. 3d–f). The capacity for erosion prevention of riparian areas is clearly higher where rivers are in good and high ecological status, but the relationship between the service and the condition is not significant for the indicators of service flow and efficiency (*p* = 0.193 and *p* = 0.923, respectively, Fig. 3g–i). The capacity and the service of flood protection and coastal protection show a positive relationship with the condition of rivers and coastal waters respectively, with higher service flow delivered by ecosystems in better ecological status (*p* < 0.05, Fig. 3j–k and 3m–n). Also the efficiency of flood protection is enhanced when the ecosystem is in good condition (*p* < 0.05, Fig. 3l). Differently, the demand of coastal protection, being related to the population density, presents an opposite relationship (*p* < 0.05, Fig. 3o). Finally, we found that the capacity for recreation increases in areas where lakes are in better ecological conditions (*p* < 0.05, Fig. 3p) and higher values for recreation (service flow) are more frequent around lakes in good and moderate ecological status (Fig. 3q).

## 4 Discussion

### 4.1 Main limitations of the indicators of ecosystem services

#### 4.1.1 Water provisioning

The WEI is a widely used indicator for water stress and its computation is straightforward. Obviously its accuracy depends on the quality of the estimation of water availability and water abstractions. In Europe several complex hydrological models are available at the continental and the river basin scale that can provide reliable estimations of the available water resources besides the Budyko equation, such as LISFLOOD (De Roo et al., 2000, De Roo et al., 2012) and PCR-GLOBWB (Sperna Weiland et al., 2010). The simple Budyko equation used here, however, has proven reliable in Europe for estimating long-term annual average water surplus availability. While the capacity of the system to provide water can be estimated by modelling, the quantification and spatial resolution of the water withdrawals for different uses entirely depends on statistics reported by countries. The latter generally do not provide the exact location and source of the abstractions, which are typically reported in aggregated form by administrative units that differ from the river basins. This implies some assumptions (and uncertainty) when spatializing the information on water abstractions. If reliable data on water abstractions are not available, other indicators can be used such as the Falkenmark index or available water per capita (Falkenmark, 1989).

#### 4.1.2 Water purification

A thorough quantification of the capacity of the aquatic ecosystems to purify water is not possible, as this is operated by microorganisms and other processes and strongly depends on local physic-chemical conditions varying in time. The estimation of nitrogen retention (both temporal and permanent) in river basins is also challenging per se; for the difficulty in quantifying nitrogen diffuse and point sources with sufficient spatial resolution, as well as the uncertainty in assessing nitrogen removal taking place in the different water bodies in the river basin, i.e. aquifers, riparian areas, rivers, lakes and estuaries. In these systems nitrogen denitrification, burial in sediments, immobilization, transformation and transport take place concurrently (Billen et al., 1991; Bouwman et al., 2013; Grizzetti et al., 2015). However, many of these processes are either represented by empirical retention coefficients in models, or require detailed information for the quantification. Examples of other models that can be used to estimate nitrogen retention are SWAT, RiverStrahler, MONERIS (Hejzlar et al., 2009; Thieu et al., 2009; Venohr et al., 2011). La Notte et al. (2017) have estimated the economic value of nitrogen retention at the European scale, using the estimations of nitrogen retention provided by the GREEN model and the replacement cost methodology. In particular, this approach takes into account the sustainability of the nitrogen retention service, introducing a threshold, based on nitrogen concentration in waters, beyond which the value of further retention starts declining.

Finally, it is important to stress that many other pollutants that are relevant for water purification are not represented by an indicator of nitrogen retention.

#### 4.1.3 Erosion prevention

The availability of spatial data on the location and type of riparian vegetation is crucial to estimate the erosion mitigation in riparian areas. The recently published data from the project COPERNICUS at the European scale represent a major step forward; however, these data do not cover smaller river stretches. A limitation of the proposed indicators of service flow and efficiency is related to the modelling of the erosion and hydrological processes, which is data and time demanding. There is a certain level of uncertainty in the representation of the processes of filtering and sediment transport in the models like SWAT. In addition, measurements of sediment needed for model calibration are often scarce, which increases the uncertainty of the predictions.

#### 4.1.4 Flood protection

The estimation of flood attenuation is based on the upscaling of equations originally developed for reservoirs and involves some simplification, such as neglecting the information on floodplain connectivity, vegetation and soil characteristics (see Supplementary material S4 for further details). Our estimate is therefore to be regarded only as an approximation, nevertheless reflecting the expected relationship between available floodplain extent and flood peak reductions.

#### 4.1.5 Coastal protection

The model-derived indicators about coastal protection show relative values, thus dependent on the study area. Although the input parameters have physical units, the final indicators must be interpreted as a ranking of coastal zones. There are large data gaps to compute these indicators, especially in the aquatic systems (e.g. appropriate seabed habitat maps from the Central and Eastern Mediterranean Sea). Also, the information about some of the input parameters is static (e.g. again, seabed habitat maps), leading to temporal assumptions. The magnitude and effects that different ecosystems have on protecting the shoreline are highly context dependent. However, this large (continental) scale analysis cannot account for the local processes, namely local sediment budget (sand availability, beach stability, etc.); subsidence; main direction of morphologic features with respect to the wave action; coastal development and management; detailed and dynamic habitat mapping with specific non-linear responses; or dynamic adaptation capability of a coastal area.

#### 4.1.6 Recreation

Except for the information on bathing water, the indicator of natural capacity to support recreation activities does not include the ecosystem condition; people are supposed to be attracted by the presence of a lake, more than the water quality. In this case, the indicator of service flow estimates a potential use of the service (i.e. potential flow of visitors). The actual number of visitors is not available at the scale of this analysis.

### 4.2 Relationship between ecosystem condition and ecosystem services

Since 2011, the Convention on Biological Diversity (through the Aichi biodiversity targets) and the EU Biodiversity Strategy have adopted the ecosystem services approach to protect biodiversity. However, understanding the relationship between the ecosystem functioning, integrity, biodiversity, and the delivery of ecosystem services is still an impellent research question (Ricketts et al., 2016; Liquete et al., 2016c; Roche and Campagne, 2017). Indeed, although there are numerous evidences supporting a positive relationship between biodiversity, ecosystem functions, and the delivery of ecosystem services (Egoh et al., 2009; Cardinale, 2011; Isbell et al., 2011; Mace et al., 2012; Harrison et al., 2014), there is not much consensus on what the links are and how they operate (Loreau et al., 2001; Harrison et al., 2014). In particular, studies at the large scale are not available.

According to the CICES classification (v4.3) followed in this study, ecosystem services are classified into three broad types: provisioning, regulating and cultural services (Table 1). For aquatic ecosystems we might expect provisioning ecosystem services to act as pressures, since they involve the extraction of products like water or fish from the ecosystem (i.e. water provisioning involves water abstraction, fish provisioning entails fish catch), implying that the higher is the provision of the service the higher is the impact on the ecosystem. On the contrary we might expect that in natural conditions regulating services, such as climate regulation, water purification and pollination, are enhanced in healthy ecosystems, with more service level provided by good ecosystem functioning. However, this might not be the case when management for ecosystem services reduces biodiversity (Adams, 2014). In addition, it is important to distinguish among the different aspects of the ecosystem services (capacity, flow, efficiency, benefits), as the service flow can be dominated by human inputs and demand. For cultural services the relationship between ecosystem services and conditions may not be straightforward. For example, the service of recreation is supported by the beauty of the natural landscape or the quality of bathing waters, but also by the presence of infrastructures and the site accessibility, and at high rates the service use contributes to the degradation of the ecosystem, due to pollution or habitat destruction.

If we consider the ecological status as an indicator of ecosystem functioning, integrity and biodiversity for aquatic ecosystems (see Section 2.2), the expected relationship between ecosystem services and ecological status might be the following: 1) *Provisioning* services are expected to have a negative relation with the ecological status (for example the more water is abstracted for human consumption the less water is available for the natural habitat for aquatic life, or high fish catches might disrupt the biodiversity or trophic chain of the aquatic ecosystem resulting in a degradation of the ecological status); 2) *Regulating* services are expected to have a positive relation with the ecological status; 3) *Cultural* services are expected to have a positive relation with the ecological status but probably to a certain limit. Similar relationships are discussed by Kandziora et al. (2013).

**Fig. 4:**
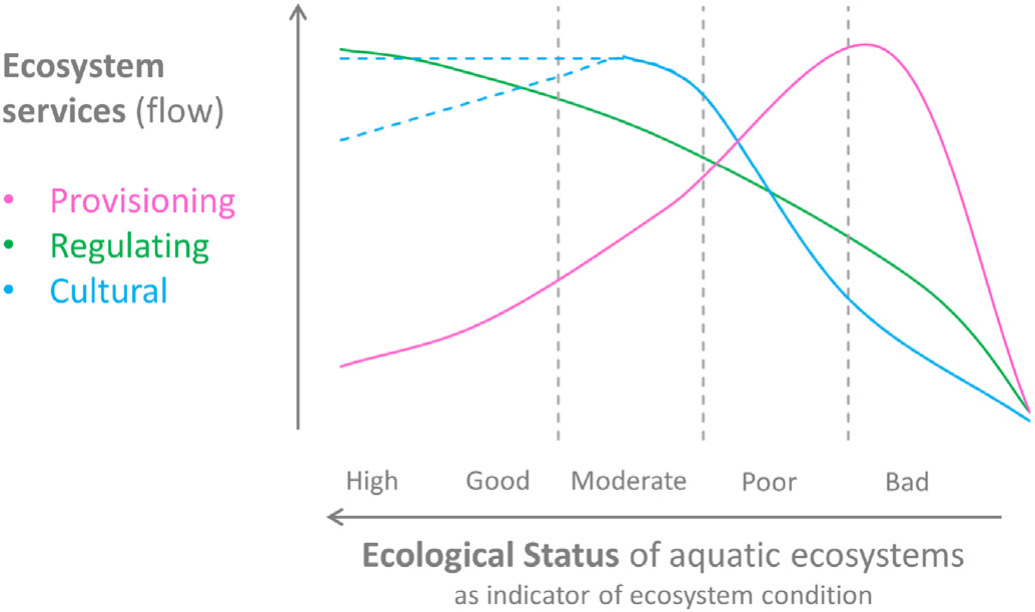
Expected relationship between the level of ecosystem services (flow) and ecological status in aquatic ecosystems.

Modified from Kandziora et al. (2013).

The expected relationship between ecosystem services and ecological status in aquatic ecosystem is shown in Fig. 4. This relationship might hold when considering indicators of the *flow* of the services (the actual use of the service). Differently, for indicators of *capacity* and *efficiency* of the services (the potential of the ecosystem to provide the service and the efficiency of the process, respectively) we expect a positive relationship with the ecological status, to indicate that good ecosystem functioning, high level of integrity and biodiversity support the capacity and the efficiency of the ecosystem to provide services. On the contrary, proxies of service demand or *benefit* should be linked to densely populated areas and thus to more degraded ecosystems (Maron et al., 2017).

Importantly, the ecological status and the ecosystem services indicators differ by theoretical definition and by the data that are used for their computation. The ecological status is an indicator of the state (condition) of the ecosystem and is evaluated at the water body scale by independent national experts. The indicators of ecosystem services are based on modelling estimates or satellite images.

Overall, the results of our analysis at the European scale based on six ecosystem services (four regulating services, one provisioning service and one cultural service) indicate that the ecosystem capacity to provide the services is always positively correlated to the ecological status (Table 3), except for water provisioning, which however strongly depends on the climatic and hydrographic characteristics of the river basin, more than on the conditions of water bodies. Indeed, water provisioning is less correlated to biodiversity compared to other ecosystem services (Harrison et al., 2014). In addition, in the present analysis we only considered the water quantity without taking into account the water quality (which is also mediated by ecosystem processes) that is required by the different uses.

**Table 3:** Relationships observed in this study between ecosystem services provided by European aquatic ecosystems and their ecological status. Expected relationships explained in Section 4.2 are reported within brackets. Legend: + indicates a positive relationship (i.e. more ecosystem service capacity/flow/efficiency or sustainability in better ecological conditions); − indicates a negative relationship (i.e. more ecosystem service capacity/flow/efficiency or sustainability in poorer ecological conditions); * indicates that the observed relationship was not significant.

	Ecosystem service indicators			
	Capacity	Flow	Efficiency or sustainability	Benefit
Provisioning				
Water provisioning	− (+)	− (−)	− (+)	
Regulating				
Water purification	+ (+)	− (+)	+ (+)	
Sediment mitigation	+ (+)	* (+)	* (+)	
Flood protection	+ (+)	+ (+)	+ (+)	
Coastal protection	+ (+)	+ (+)		− (−)
Cultural				
Recreation	+ (+)	+ (+)		(−)

From the analysis we observe that provisioning services (considering the indicators of service flow) are correlated with lower ecological status, suggesting that they act as pressures for the aquatic ecosystems, i.e. their increase degrades the ecosystem functioning, although a causal relationship cannot be proven by our analysis. Similarly, benefits/demand decrease with improved ecological status, mainly due to the population density (see for example coastal protection). On the contrary, flow and efficiency indicators of the regulating services of flood protection and coastal protection increase with better ecological status. Differently, in the case of water purification flow, the indicator nitrogen retention is related to human input of nitrogen pollution to rivers and lakes. The more nitrogen is discharged to the water bodies, the more nitrogen is removed by the ecosystem, but the efficiency of the service (efficiency indicator) decreases with worsening of ecosystem conditions. For cultural services, our analysis shows that recreation is higher where lakes are in better ecological status, with a change in behaviour already starting for lakes in moderate status. The latter is in line with the findings of Reynaud et al. (submitted), who assessed the economic value of ecosystem service provided by European lakes. Similar evidence was reported by Pouso et al. (2018b) for the Nerbioi-Ibaizabal estuary in North Spain.

Our results are in line with the recent findings for terrestrial ecosystems of Smith et al. (2017), who carried out a systematic review of the links between ecosystem characteristics and ecosystem services. They showed that regulation and cultural services have mainly positive links with biotic attributes, such as habitat, diversity, species or functional groups, while water provisioning presents a negative link. In addition, they highlighted some bundles of ecosystem services influenced by similar natural capital attributes and thus having an analogous delivery depending on ecosystem conditions. Among others these bundles consist of 1) regulating services including water purification, flood protection and erosion protection; 2) water provisioning; and 3) cultural services, as observed in this study. Similarly and in agreement with the results of our study, Lee and Lautenbach (2016) found that there are mainly synergistic^1^ relationships among regulating services, while provisioning services tend to show trade-offs with regulating services, and cultural services are mainly correlated with regulating services.

### 4.3 Implication for policy actions

Nature supports human well-being but in turn intensive human activities produce pressures on the ecosystems, altering their conditions. Degraded aquatic ecosystems can lose their capacity to provide services (Keeler et al., 2012). These tight connections between socio-economic drivers, pressures on the environment, alteration of ecosystems biodiversity and functioning, and delivery of ecosystem services (provisioning, regulating and cultural) are at the core of sustainable development policies and environmental regulations that seek to achieve sustainable management of water and natural resources.

In this study we focused on the relationship between ecosystem services and ecosystem condition. In addition, while this study provides a picture of the current situation in Europe, the proposed indicators could also be used to assess changes in time, as the estimation of ecosystem services (service flow) is mainly based on models that can be used in scenario analysis (Guswa et al., 2014).

Quantifying the ecosystem services, as in this study, and identifying when they correspond also to pressures, such as the case of water provisioning or recreation in highly populated areas, help in understanding the value of protecting ecosystems for human society. Importantly, showing evidence of the link between good ecosystem conditions and higher provision of ecosystem services justifies the effort and the cost of maintaining ecosystems in good conditions or restoring them (Acuña et al., 2013). Although we cannot conclude that there is a causal relationship, our analysis limited to six ecosystem services (four regulating services, one provisioning service and one cultural service) indicates that aquatic ecosystems in better ecological status are generally correlated with higher delivery of ecosystem service. It should be noted that including more provisioning services or other services in the analysis might have provided different results.

## 5 Conclusion

The analysis of the relationship between aquatic ecosystem conditions, as defined by class of Ecological Status of the EU legislation (Water Framework Directive), and the metrics of ecosystem services (capacity, flow, sustainability, efficiency, benefit) done in this study is relevant to understand how the protection and restoration of the aquatic ecosystems support human well-being and how the exploitation of ecosystems can affect the conditions of the aquatic environment.

Our study indicated that regulating and cultural water ecosystem services are mostly positively correlated with the ecological status of European water bodies, suggesting that more service is provided by an ecosystem in good condition. We also shed light on the role of provisioning services, distinguishing between indicators that describe their action as pressures (flow), and those describing the capacity and sustainability of the service.

Our results, although limited to the ecosystem services analysed, show the relevance of water ecosystem services to sustain human activities and well-being, and the importance of maintaining good ecological conditions of aquatic ecosystems to ensure the delivery of ecosystem services in the future. This evidence is relevant to consider when investing in water ecosystems conservation and restoration as called for by the current EU water policy and Biodiversity Strategy, and more generally provides scientific ground to support actions implementing SDG6 and SDG15 and the Convention on Biological Diversity at the global scale.

## Declarations of interest

None.

## Appendix A Supplementary data

Supplementary material Image 1

## Footnotes

1 Lee and Lautenbach (2016) used the term “synergistic” when two services change positively in the same direction.
