# Supplementary material for: Relationship between ecological condition and ecosystem services in European rivers, lakes and coastal waters
Source: Sci Total Environ. 2019 Jun 25;671:452–65. doi: 10.1016/j.scitotenv.2019.03.155 (PMC6509285; doi:10.1016/j.scitotenv.2019.03.155)
Supplement: Supplementary file 1 — Supplementary material [file mmc1.docx]

# Supplementary Material

## S1. Catchments number and area distribution

| 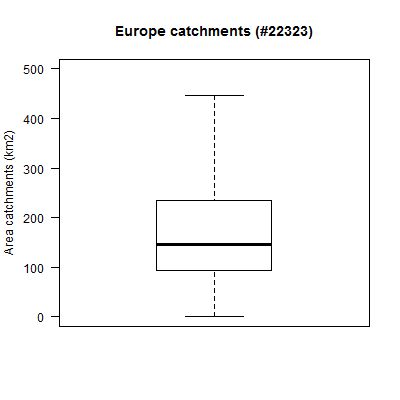 | 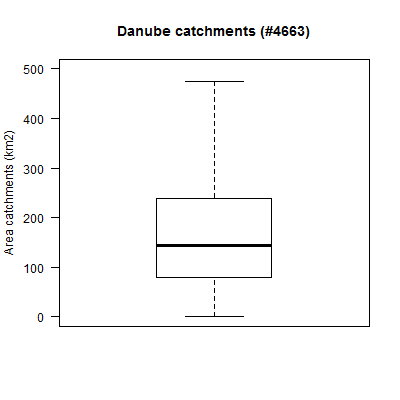 |
| --- | --- |

Figure S1 Area distribution of the catchments adopted for the assessment of ecosystem services and ecological status for rivers and riparian areas for Europe (left) and in the Danube river basin (right). The number of catchments is indicated in the title within brackets.

## S2. Glossary of terms adopted in the study

|  | **Definitions and references** |
| --- | --- |
| **Benefits** | *“The direct and indirect outputs from ecosystems that have been turned into goods or experiences that are no longer functionally connected to the systems from which they were derived. Benefits are things that can be valued either in monetary or social terms. [OpenNESS]*” (Potschin et al. 2016) |
| **Ecosystem Condition** | *“Ecosystem condition is the physical, chemical and biological condition or quality of an ecosystem at a particular point in time”* (Maes et al. 2018)  *“Ecosystem condition reflects the overall quality of an ecosystem (or ecosystem asset), in terms of its main characteristics underpinning its capacity to generate ecosystem services. Ecosystem condition should be measured by indicators representing the quality of its key components (such as water, soil, biodiversity, nutrient flow, or landscape configuration) with respect to a reference condition”.* (Potschin et al. 2016)  See also Roche and Champagne (2017) for a discussion on the notion of ecosystem integrity (ecosystem condition, ecosystem state, ecosystem health) |
| **Ecosystem Service Bundle (supply side)** | *“A set of associated ecosystem services that are linked to a given ecosystem and that usually appear together repeatedly in time and/or space”.* (Potschin et al. 2016) |
| **Ecosystem Service Demand** | *“The amount of a service required or desired by society [Based on Villamagna et al. (2013)]”* (Potschin et al. 2016)  See also Villamagna et al. (2013) |
| **Ecosystem Service Flow** | *“A measure for the amount of ecosystem services that are actually mobilized in a specific area and time. It includes a dynamic temporal dimension and conceptually links ecosystem service supply with demand. [ESMERALDA]*” (Potschin et al. 2016)  See Grizzetti et al. (2016) for indicators of ecosystem service flow |
| **Ecosystem Service Supply** | *“The provision of a service by a particular ecosystem, irrespective of its actual use. It can be determined for a specified period of time (such as a year) in the present, past, or future. [ESMERALDA]”* (Potschin et al. 2016) |

**References**

ESMERALDA, 2019. European Union's Horizon 2020 research and innovation programme under grant agreement No 642007. <http://esmeralda-project.eu/> (accessed in January 2019).

Grizzetti, B., Lanzanova, D., Liquete, C., Reynaud, A., Cardoso, A.C., 2016. Assessing water ecosystem services for water resource management. Environmental Science and Policy 61, 194-203.

Maes J., Teller A., Erhard M., Grizzetti B., Barredo J.I., Paracchini M.L., Condé S., Somma F., Orgiazzi A., Jones A., Zulian A., Petersen J.E., Marquardt D., Kovacevic V., Abdul Malak D., Marin A.I., Czúcz B., Mauri A., Loffler P., Bastrup-Birk A., Biala K., Christiansen T., Werner B., 2018. Mapping and assessment of Ecosystems and their Services: An analytical framework for ecosystem condition. Publications office of the European Union, Luxembourg.

OpenNESS, 2019. European Union’s Seventh Programme for research, technological development and demonstration under grant agreement No 308428. <http://www.openness-project.eu/> (accessed in January 2019).

Potschin, M., Haines-Young, R., Heink, U., K. Jax, 2016. OpenNESS Glossary (V3.0), 39 pp. Grant Agreement No 308428. Available from: <http://www.openness-project.eu/sites/default/files/OpenNESS_Glossary_final.pdf> (accessed in January 2019).

Roche, P.K., Campagne, C.S., 2017. From ecosystem integrity to ecosystem condition: a continuity of concepts supporting different aspects of ecosystem sustainability. Current Opinion in Environmental Sustainability 29, 63-68.

Villamagna, A.M.; Angermeier, P.L. and E.M. Bennett (2013): Capacity, pressure, demand, and flow: A conceptual framework for analyzing ecosystem service provision and delivery. Ecol. Complex 15: 114–121.

## S3. Mapping and assessment of fish provisioning in Europe

Food provision is the delivery of biomass for human consumption and the conditions to grow it. Regarding freshwater habitats, it relates mainly to inland fishing activities. In Europe, we can neglect subsistence fishing and recreational fishing as relevant sources of food, and concentrate on commercial fisheries. They can rely on wild fisheries and on aquaculture (mainly fish harvesting).

*Natural capacity* - The ecological assessment of fish populations (diversity, stock, species condition, etc.) can be used to characterise the ecosystem capacity to supply fish for human consumption. We considered the “Composition, abundance and age structure of fish fauna”, which is one of the biological quality elements (BQE) under the WFD, i.e. a sub-indicator of the overall ecological status (Figure S3a). Other possible indicators that could be used are (1) the conservation status of freshwater fish species of community interest under the EU Habitats Directive (Directive 1992/43/EEC), but the relevant information available is less explicit in geographical terms and the latest Member States reporting (of 2013) is still not available as a European database; (2) an ecological assessment (comparable to the conservation status of freshwater fish species of community interest) developed by Freyhof and Brooks (2011), still lacking detailed geographical representation; or (3) specific stock assessments of commercial species, usually developed at regional level and not publicly available.

*Service flow -* The total production from freshwater fisheries, differentiating between wild fisheries and aquaculture production, represents the flow or delivery of food provision. The best source of information for a European scale analysis is the EUROSTAT Fisheries database^[[1]](#footnote-1)^ which holds data on inland fisheries production from catches and aquaculture at national level, but with different time coverage between 1998 and 2011 (Figure S3b). The FAO global fisheries database (FishStatJ^[[2]](#footnote-2)^) compiles similar data from 1950 to 2010, making it more suitable for long-term analyses. Additional sources of information could be Mitchell et al. (2012) for national statistics or the FishBase^[[3]](#footnote-3)^ for species details.

*Sustainability or efficiency* - An ideal sustainability indicator of food provision would compare the wild captures (flow) *vs*. quantity of adult, healthy commercial fish (capacity). However, the lack of temporal coverage of our capacity indicator and the lack of spatial resolution of our flow indicator makes it impractical to compare both proxies. As an alternative, one can analyse the temporal trend of the freshwater fish catches focusing on the evolution of wild fisheries, since aquaculture production depends more on human inputs and industrial decisions than on natural capacity. The trend analysis of freshwater fish captures in EU, candidate and Balkan countries (34 countries) represented in Figure S3c was based on 20 long-term series (with data since the 50s or 60s) and 14 shorter data series from the FishStatJ database.

*Benefit* - We can propose some suitable indicators to value this service such as the market value of the fish catch (differentiating between public and private investments, or industrial benefits and subsidies, which can be particularly important in this sector); the employment generated by the fishing activities; or the portion of freshwater fish in the human diet composition. For monetary values, possible data sources could be the EUMOFA^[[4]](#footnote-4)^ database, the GLOBEFISH^[[5]](#footnote-5)^ market reports, or specific publications (e.g. Tveterås et al. 2012).

*Main limitations of the indicators -* For the service food provisioning the results must be considered preliminary. The low spatial resolution of the public data available (e.g. fisheries information only at country level) and the number of data gaps found (e.g. lack of monitoring or reporting of the Good Ecological Status’ sub-indicators, such as the status of the composition of fish communities, in many water bodies) hamper the usefulness of these results. However, the proposed metrics could be applied and could generate significant results at a local scale, provided that more detailed information is available. In addition, the indicator “Composition, abundance and age structure of fish fauna” is quite ambiguous and qualitative. More specific quantitative metrics coming from monitoring networks or modelling approaches would be preferred. The analysis of caches trends is shown as a proxy of the state of fish stocks but it also relates to multiple natural and economic factors, so it cannot be interpreted as a direct sustainability index. An alternative could be for instance the ratio between wild fisheries and total population biomass.

*State of the service in Europe -* The capacity of European freshwaters to support wild fisheries is largely unknown at the scale of this analysis and varies among countries, although fish in lakes seem to be in better conditions. The decrease of fish catch is evident in most European countries at least since the 1980s, most probably due to the health and biomass decline of fish populations. Aquaculture production is increasing only in countries where its relative importance (total production) is still low. The most common trend, observed in 24 countries, is a continuous rise in captures until reaching a maximum peak, usually occurring during the 80s, followed by a more or less sudden drop until today.

| **Food provisioning** | | |
| --- | --- | --- |
| a. Natural capacity | b. Service flow | c. Sustainability |
| 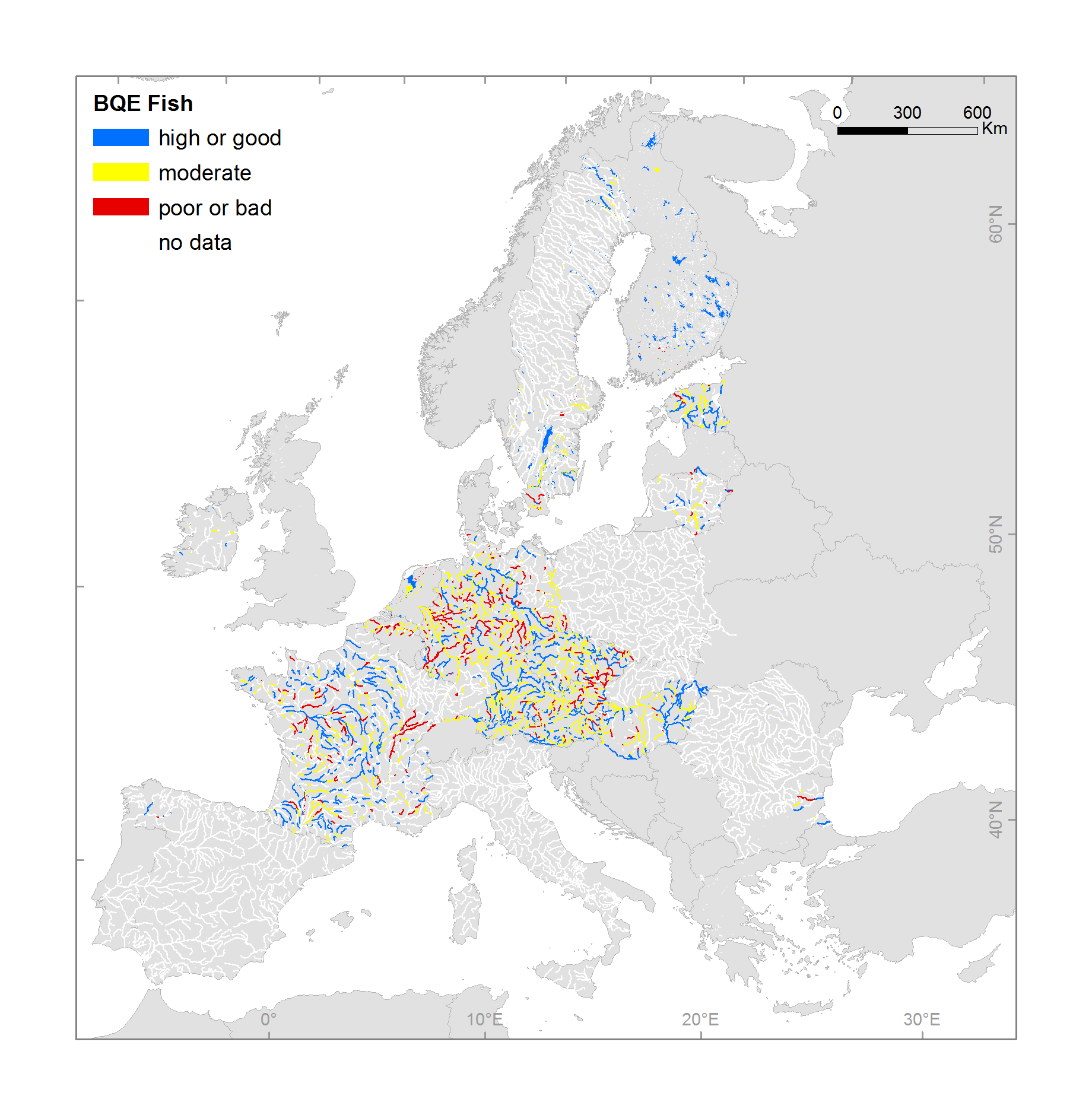 | 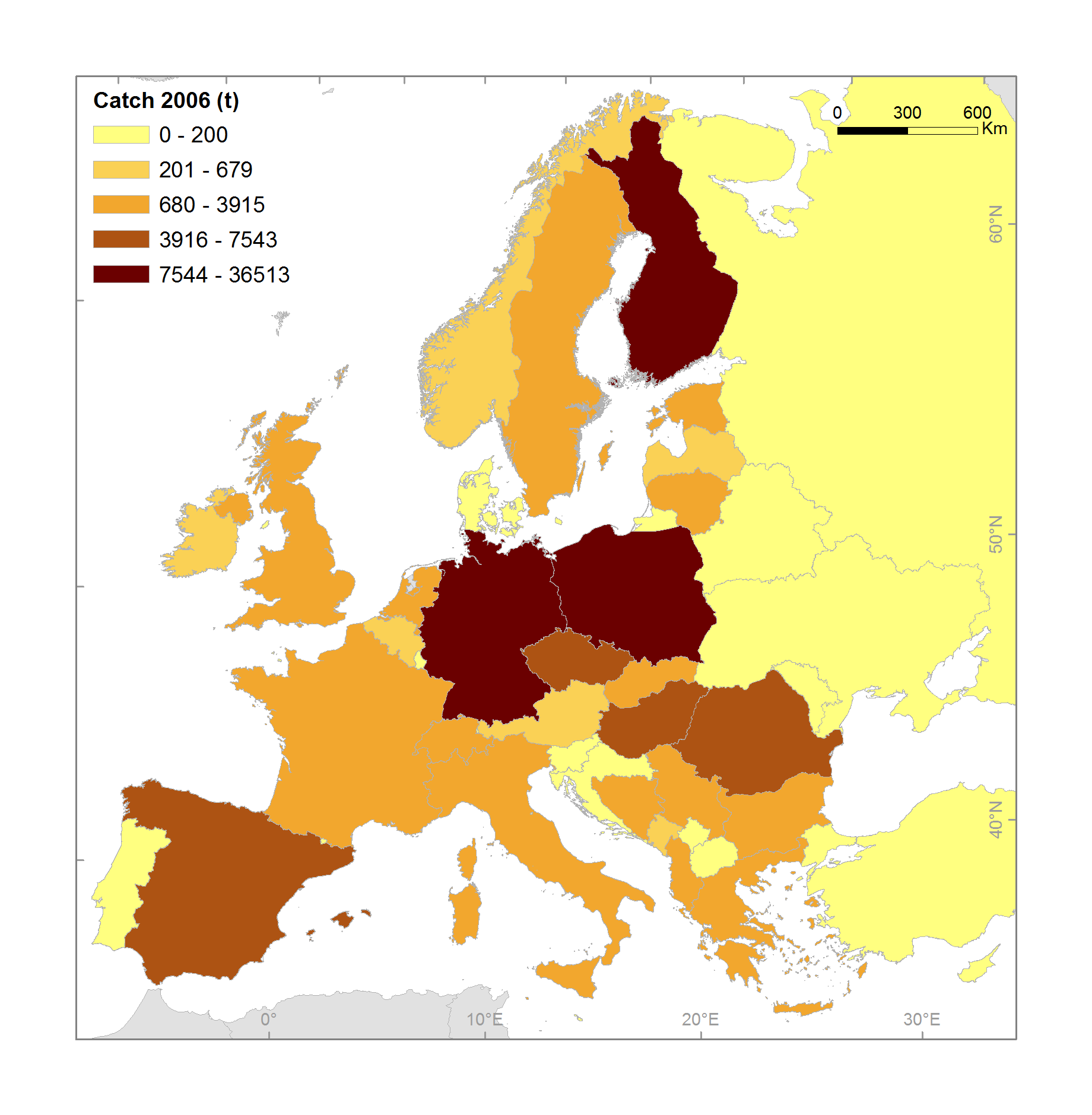 | 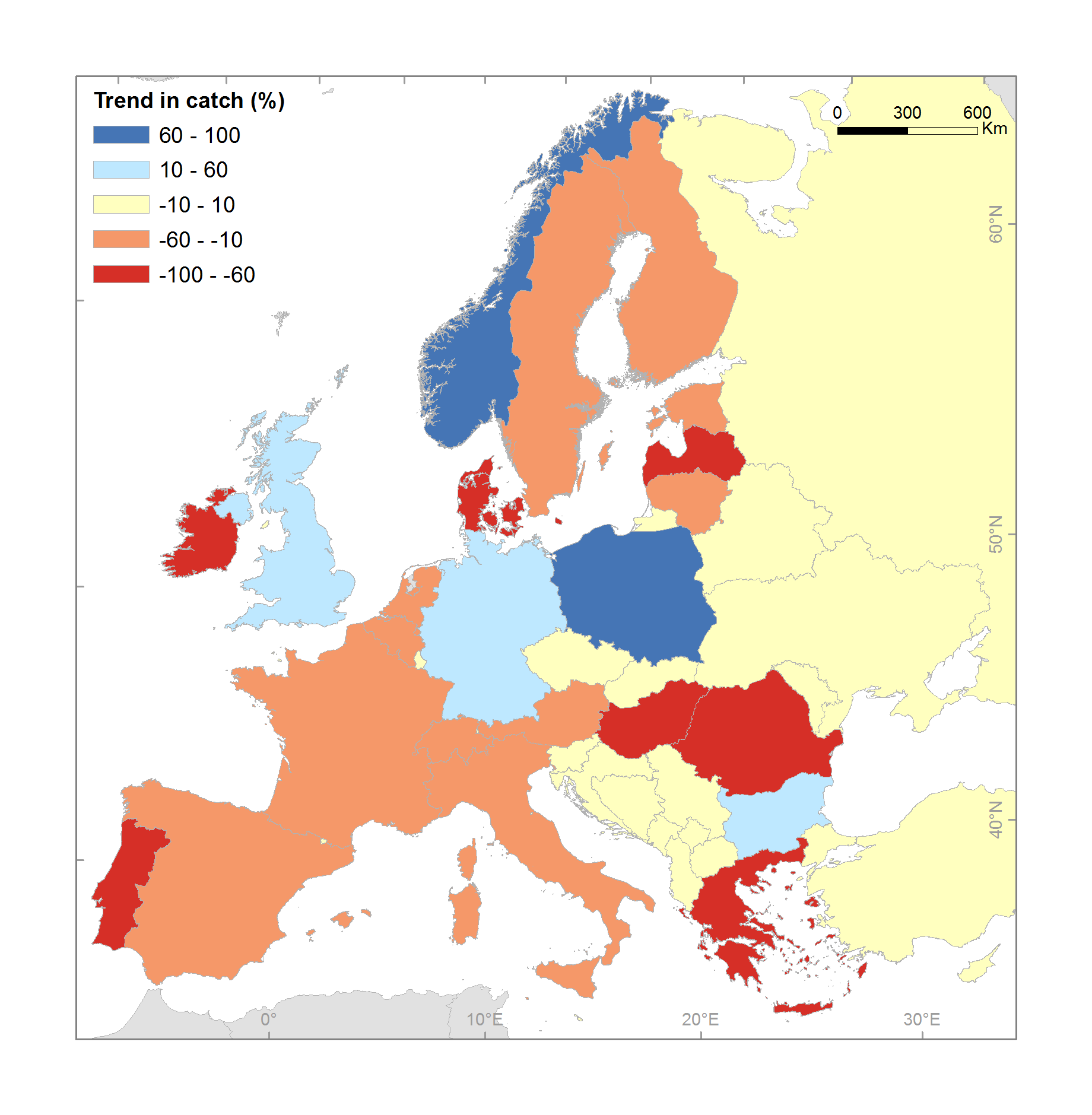 |

**Figure S3** European maps of the ecosystem service food provisioning (in rivers and lakes): **a.** Fish ecological status (sub-indicator Fish of the WFD ecological status) reflecting the composition, abundance and age structure of fish per surface water body. This is selected as an indicator of the natural capacity to provide fish. **b.** Wild captures (catch) from inland fisheries (in tonnes live weight for the year 2006) reported by Member States and collected in the EUROSTAT Fisheries database. One value per country is the maximum resolution at which these data are available. **c.** Trend in wild captures or catch (from the 1980s to today) from inland fisheries as reported by FAO.

**References**

Freyhof, J. and Brooks, E., 2011. European Red List of Freshwater Fishes. IUCN. Luxembourg: Publications Office of the European Union.

Mitchell, M., Vanberg, J., Sipponen, M., 2012. Commercial inland fishing in member countries of the European Inland Fisheries Advisory Commission (EIFAC). Operational environments, property rights regimes and socio-economic indicators. Country profiles 2010. EIFAC-FAO.

Tveterås S, Asche F, Bellemare MF, Smith MD, Guttormsen AG, et al., 2012. Fish Is Food - The FAO’s Fish Price Index. PLoS ONE 7(5): e36731.

## S4. Methodology to estimate flood protection

*Service flow* - In a catchment, if the whole floodplain were left natural, the flood peak reduction can be quantified at a screening level on the basis of a simple model following Marone (1971):

$\eta=\frac{Qout}{Qin}=\max\left( 0, 1- \frac{Ah}{W} \right)$ (Eq. 1)

where Qout is the peak flood discharge of the hydrograph downstream of the floodplain; Qin is the discharge of the hydrograph upstream the floodplain; A is the area of the floodplain; h is the average depth of water in the floodplain at the peak of discharge; and W is the flood hydrograph volume. We present here a tentative quantification of this indicator for Europe, using the results of flooding simulations from Alfieri et al. (2014). In particular, we use the flood hydrograph volume W as defined therein, as well as the flooded area extent A and the flood peak discharge Qout. The flood extent is mapped through Boolean map as grids with a resolution of 1 ha, where 1 means presence of flooding and 0 its absence. We assume that, in the absence of flooding, the flood peak discharge estimated by the authors would be higher and equal to Qin.

In order to compute the average depth h, we make use of Manning’s formula in the form:

$h=\left( \frac{n Qout}{B\sqrt{J}} \right)^{0.6}$ (Eq. 2)

where n is the roughness coefficient of the floodplain (that we assume equal to 0.1 s m^-1/3^), B is the width of the floodplain orthogonal to the flood propagation direction, and J the slope of the floodplain.

We compute the indicator $\eta$ with reference to 5-km long stretches of the main European stream network (corresponding to rivers with a drainage area of 500 km^2^ or more). For each of these stretches, estimates of Qout are available following Alfieri et al. (2014). For this exercise, we carried out the analysis for floods with a return period of 200 years. We compute Thiessen polygons (nearest neighbour regions) for each 5-km stretch, and we consider the extent of flooded area within each Thiessen polygon, yielding parameter A in Equation 2. Dividing A by the length of the stretch (5 km) yields an estimate of the average width, B. Slope J is assigned to the river stretch as the average of values estimated at 1 km resolution for Europe, as described in Pistocchi et al. (2007). With these assumptions, we obtain the indicator $\eta$ from Equation 1.

The flow peak attenuation (m^3^/s) per catchment is computed as:

$Attenuation=Q_{out}*\frac{1-\eta}{\eta}$ (Eq. 3)

This reduction of flood peak discharges would represent the maximum theoretical attenuation of the floodplain if all area were inundated. However, in Europe large parts of floodplains have been occupied by agricultural lands and urban areas. Flooding of a natural floodplain, in principle, does not entail any damage as this is an area structurally fit to allow this hydrological process. But in many floodplains, in order to protect settlements and, less frequently, agricultural land, flooding is prevented by man-made defences.

The actual service flow for flood protection can be described as the flood attenuation allowed only by floodplains in natural conditions in presence of a flood event. A first order quantification of this attenuation can be computed by using Equation 1 and Equation 3, where the total flooded area A is computed as the total areas of the floodplain minus the urban areas and part of agricultural areas (Attenuation URB). We used the Corine Land Cover (CLC)^[[6]](#footnote-6)^ (year 2012) to identify urban land and agricultural land in floodplains, considering the CLC classes “Artificial surfaces” (class 1) and “Agricultural areas” (class 2) respectively. We overlaid the information on land cover with the flood extent maps produced by Alfieri et al. (2014), considering floods with return time of 200 years and artificial flood defence of agricultural areas of 50%. With lower A, application of Equation 1 yields higher values of η, i.e. lower attenuation of the flood peaks.

*Main limitations of the indicator* - The calculation is to be regarded as preliminary and indicative. The model of Equation 1 was developed by Marone (1971) with reference to reservoirs for the attenuation of floods. Although the author developed the model through numerical experiments that may be referred also to the case of floodplains, the assumptions made by the author are extremely simplified and are not necessarily realistic for many real world situations.

**References**

Alfieri, L., Salamon, P., Bianchi, A., Neal, J., Bates, P. and Feyen, L., 2014. Advances in pan-European flood hazard mapping. Hydrol. Process., 28: 4067–4077. doi:10.1002/hyp.9947

Marone, V., 1971. Calcolo di massima di un serbatoio di laminazione. L’Energia Elettrica, n. 9, pp 561-567. In Italian.

Pistocchi, A., Vizcaino, M.P., Pennington, D.W., 2007. Analysis of Landscape and Climate Parameters for Continental Scale Assessment of the Fate of Pollutants EUR 22624 EN ISSN: 1018-5593 ISBN: 978-92-79-04809-8. Luxembourg: Office for Official Publications of the European Communities

1. <http://epp.eurostat.ec.europa.eu/portal/page/portal/fisheries/data/database> [↑](#footnote-ref-1)
2. <http://www.fao.org/fishery/statistics/software/fishstatj/en> [↑](#footnote-ref-2)
3. <http://www.fishbase.org/search.php> [↑](#footnote-ref-3)
4. <http://ec.europa.eu/fisheries/market-observatory/home> [↑](#footnote-ref-4)
5. <http://www.globefish.org/homepage.html> [↑](#footnote-ref-5)
6. See <http://land.copernicus.eu/pan-european/corine-land-cover> [↑](#footnote-ref-6)
